# Mobile Health Apps for Pregnant Women: Systematic Search, Evaluation, and Analysis of Features

**DOI:** 10.2196/25667

**Published:** 2021-10-18

**Authors:** Gabriela Frid, Kelly Bogaert, Katherine T Chen

**Affiliations:** 1 Icahn School of Medicine at Mount Sinai New York, NY United States

**Keywords:** app, app store, mHealth, mobile health, prenatal, pregnancy, women's health

## Abstract

**Background:**

Many pregnant women use the internet to obtain information about pregnancy and childbirth. Over 50% of pregnant women use pregnancy apps and must search through thousands of pregnancy or women’s health–related apps available on app stores. The COVID-19 pandemic is changing how women receive prenatal care. Mobile health apps may help maintain women’s satisfaction with their prenatal care.

**Objective:**

Our objective is to identify pregnancy mobile apps and to evaluate the apps using a modified APPLICATIONS (app comprehensiveness, price, privacy, literature used, in-app purchases, connectivity, advertisements, text search field, images/videos, other special features, navigation ease, subjective presentation) scoring system.

**Methods:**

A list of pregnancy apps was identified in the first 20 Google search results using the search term “pregnancy app.” After excluding irrelevant, inaccurate, malfunctioning, or no longer available apps, all unique apps were downloaded and evaluated with the modified APPLICATIONS scoring system, which includes both objective and subjective criteria and evaluation of special features.

**Results:**

A list of 57 unique pregnancy apps was generated. After 28 apps were excluded, the remaining 29 apps were evaluated, with a mean score of 9.4 points out of a maximum of 16. The highest scoring app scored 15 points. Over 60% (18/29) of apps did not have comprehensive information for every stage of pregnancy or did not contain all four desired components of pregnancy apps: health promotion/patient education, communication, health tracking, and notifications and reminders. Only 24% (7/29) of apps included a text search field, and only 28% (8/29) of apps cited literature.

**Conclusions:**

Our search yielded many high-scoring apps, but few contained all desired components and features. This list of identified and rated apps can lessen the burden on pregnant women and providers to find available apps on their own. Although health care providers should continue to vet apps before recommending them to patients, these findings also highlight that a Google search is a successful way for patients and providers to find useful and comprehensive pregnancy apps.

## Introduction

The internet and smartphones are increasingly popular both as a means to access health care information and as tools for health care management [1]. During the COVID-19 pandemic, in-person health care visits dropped and patients sought care via different methods [2]. Recent data shows that 21% of prenatal visits in March 2020 were impacted (ie, cancelled, rescheduled, or conducted remotely) [3]. In April 2020, 26% of prenatal visits were impacted [3]. For those that had remote phone or video visits, less than half felt they received the same amount or more information and care as compared to that received during a traditional visit [3]. The pandemic has also caused a 25% increase in mobile health app downloads as compared to the same time last year [4].

Internet use during pregnancy is common [5]. The majority of women in the United States own a smartphone and past studies have shown that over 50% of pregnant women download apps focused on pregnancy, downloading an average of three during the prenatal period [6-8]. Women who use mobile prenatal care apps and have fewer prenatal visits have no reduction in patient satisfaction [9]. Health care–related apps account for a large market share of the apps available on the US iOS App Store, with almost 100,000 apps as of 2017 [10]. A 2013 study showed that pregnancy apps are the most used health apps [11]. Although 94% of pregnant women reported that a smartphone changed their lives for the better, there are many apps to choose from and the market for apps is constantly growing [6].

Due to the number of apps available to pregnant women, knowing what apps are available, how to select among them, and what information and features women are seeking is important. Wang et al [12] surveyed 535 women and showed that the most common reasons women used apps were to monitor fetal development (83%) and to obtain information on nutrition (26.2%) and antenatal care (23.9%). Lee et al [8] evaluated 47 apps that were identified by surveying 193 women and reported that most women decided to download an app after evaluating its content themselves (61.7%), and that the most frequently cited benefit was convenience (35.8%), while the most common weakness was lack of credibility (39%).

These studies provide information about why and how women use apps, but there have been no critical quantitative analyses done to evaluate and rate these apps in terms of their usefulness and benefit to pregnant women. The purpose of this study is to identify and evaluate pregnancy apps recommended to women on the internet. Our study additionally sought to create a comprehensive list of app features and a quantitative measure of comprehensiveness.

## Methods

This study did not require review by the Institutional Review Board at the Icahn School of Medicine at Mount Sinai as it did not involve human subjects. We used the web search engine developed by Google, using the search term “pregnancy app” in March 2019. Search engines and online reading are top ways that consumers discover apps, and we selected Google’s search engine because it accounts for over 90% of all search queries [13,14]. The first page, with an average of 10 search results, accounts for 91.5% of traffic, while the second page accounts for 4.8% [15]. We confirmed that the results displayed were nearly identical when searched through different devices (eg, computer, iPad, and iPhone) and from different geographical locations. Google Incognito mode was used to limit variation of results based on the user’s browser cookies.

We downloaded all apps mentioned within the first 20 search results. We noted characteristic information—such as app name, app store, and app icon—and evaluated app content using an adapted APPLICATIONS (app comprehensiveness, price, privacy, literature used, in-app purchases, connectivity, advertisements, text search field, images/videos, other special features, navigation ease, subjective presentation) scoring system. APPLICATIONS is an acronym for components of apps that can be scored to help determine the rating of the app [16]. The usefulness and benefit of the apps was determined by the app comprehensiveness score, which judges the ability of an app to provide useful pregnancy information, and by the other components of the APPLICATIONS scoring system, which judge other aspects that have been shown to make apps successful [16]. This score was devised based on availability of information on the four distinct portions of pregnancy care, as well as the top four functions of the most commonly used pregnancy apps, as per Lee et al [8].

Other modified components of the original APPLICATIONS scoring system are described here. “Price” and “Paid subscription” were combined into one “Price” score. “Privacy” was added because while the initial APPLICATIONS scoring system was created with providers in mind, this study is evaluating patient-facing apps, and patients have expressed security and privacy concerns as an important consideration when choosing whether to use an app [17]. “Interdevice compatibility” was removed as each app denotes the proper platform to use. “Images/videos” was added, which was part of “Other components” previously. The “Other components” category was therefore renamed “Other special features” and the scoring was expanded due to the numerous special features provided on pregnancy apps, as the initial APPLICATIONS scoring system was intended for pregnancy wheel dating apps, which serve a more targeted, specific function and are less likely to have numerous other features.

One author (GF) checked the apps in the app stores to see if there were in-app purchases and also downloaded and opened the apps in airplane mode to evaluate the connectivity component and determine if functionality was dependent on internet access. All three authors evaluated the remaining features using the APPLICATIONS scoring system, as shown in Table 1. App comprehensiveness was determined as shown in Textbox 1. The “Other special features” category was tabulated in Multimedia Appendix 1. Navigation ease and subjective appearance were scored using a Likert scale with 1=poor, 2=below average, 3=average, 4=above average, and 5=excellent.

**Table 1:** The APPLICATIONS scoring system [16,17].

Component	Maximum score	Description
App comprehensiveness	3	0=none, 1=1-2 components, 2=3-5 components, 3=6-8 components
Price	1	0=priced, 1=free
Privacy	1	0=none, 1=privacy statement or login
Literature used	1	0=no references, 1=references used
In-app purchases	1	0=present, 1=absent
Connectivity	1	0=internet required, 1=internet not required
Advertisements	1	0=present, 1=absent
Text search field	1	0=no search field, 1=search field present
Images/videos	2	0=absent, 1=images or videos, 2=images and videos
Other special features	2	0=absent, 1=1-4 special features, 2=5-9 special features
Navigation ease	1	0=ease of navigation score <3, 1=ease of navigation score ≥3
Subjective presentation	1	0=subjective presentation score <3, 1=subjective presentation ≥3
Total	16	Sum of all scores

**App comprehensiveness criteria [8].**

Criteria:

- Health promotion/patient education
- Patient communication
- Health tracking
- Notifications and reminders
- Preconception information
- Antepartum information
- Intrapartum information
- Postpartum information

Scoring: 0 points if no components were present, 1 point for 1-2 components, 2 points for 3-5 components, and 3 points for 6-8 components.

To account for any interobserver differences, we reconciled ratings and recorded objective errors. Our reconciliation process included a meeting of all authors during which we discussed each feature of each app. When there was a discrepancy, we determined whether it was a transcription or misclassification mistake and arrived at 100% consensus for the objective components of each app. For navigation ease and subjective presentation, we averaged reviewers’ scores and awarded 0 points for an average rating of <3 and 1 point for an average rating of ≥3. We subsequently calculated a final total score for each app.

## Results

The first 20 search results from the first two pages of a Google search on a computer for the term “pregnancy app” were recorded. The results were either specific apps (n=4) or articles about apps (n=16). All the apps listed in each result were noted and this search yielded 57 apps.

**Figure 1:**
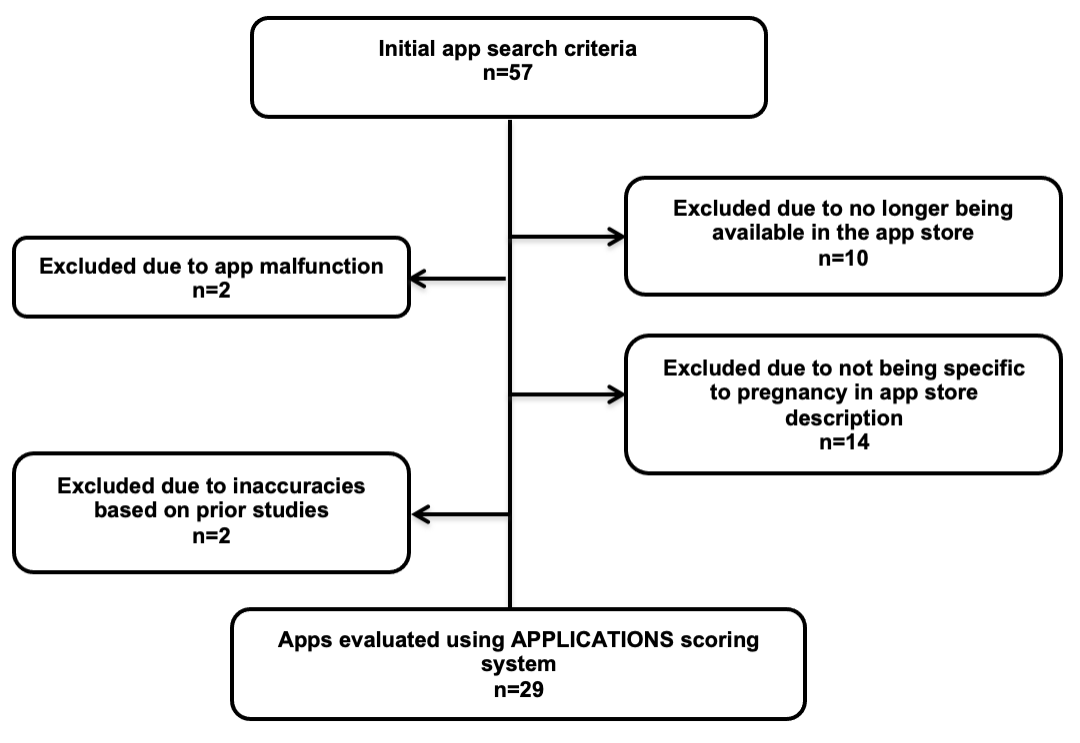
Google search for pregnancy apps. APPLICATIONS: app comprehensiveness, price, privacy, literature used, in-app purchases, connectivity, advertisements, text search field, images/videos, other special features, navigation ease, subjective presentation.

A total of 28 apps were excluded for the following reasons (Figure 1): 12 apps were no longer available or did not work, 14 apps were not related to pregnancy based on not having the word “pregnancy” in the description on the app store, and 2 were deemed inaccurate based on prior studies [18,19]. The remaining 29 apps were all downloaded on the Apple App Store or Google Play Store and evaluated between July-November 2019.

The 29 remaining apps were scored using the APPLICATIONS scoring system by all authors (Multimedia Appendix 1). The highest scoring app earned 15 out of 16 possible points. In addition, 11 apps scored above the mean score of 9.4. The lowest scoring app earned 4 points.

We determined that 41% (12/29) of apps included ≥6 comprehensiveness components out of 8, while 24% (7/29) of apps only included 1-2 comprehensiveness components out of 8, signifying missing information with regard to all stages of pregnancy or desired functionalities of pregnancy apps: health promotion/patient education, communication, health tracking, and notifications and reminders.

**Figure 2:**
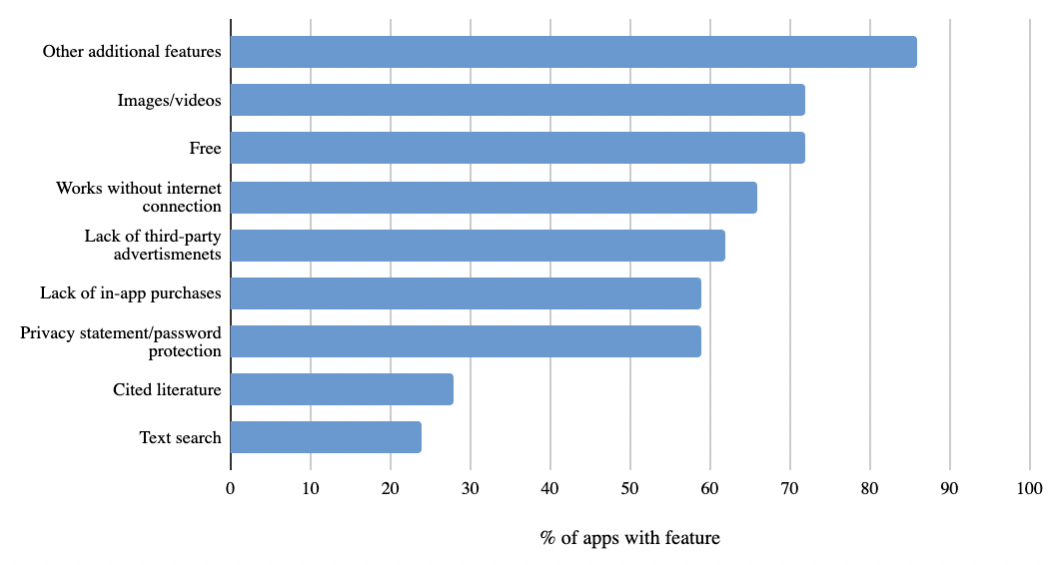
Features and functionalities of apps.

Evaluated features and functionality are shown in Figure 2. Common features and functionality included special features (25/29, 86%), free cost (21/29, 72%), images and/or videos (21/29, 72%), offline functionality (19/29, 66%), including a privacy statement or password protection (17/29, 59%), lack of third-party advertisements (18/29, 62%), and lack of in-app purchases (17/29, 59%). Use of cited literature (8/29, 28%) and text search (7/29, 24%) were rare features.

The objective component reporting error rate was 31 of 435 (7.1%), meaning that the authors independently assigned the same score to a given objective component of an app 92.9% of the time.

## Discussion

### Principal Results

In this study, we simulated how pregnant women would find apps by performing a Google search and then we evaluated and rated the identified apps. Although our results present several high-scoring apps, few contain all the components and features that make up an accurate and comprehensive app. More research is needed to survey patients and providers about which features are the most highly desired and needed. Many apps were missing cited sources, making it difficult to interpret accuracy. The rarest component was a text search, which can make it difficult for women to sift through information and quickly find what they are looking for. The most common app-specific features were contraction timers, journaling/photo uploads, and appointment trackers, checklists, and calendars. The least common were tools for obtaining safety information and health/fitness, despite these being common reasons pregnant women seek out apps [12].

### Comparison With Prior Work

Prior studies have evaluated apps by asking pregnant women directly what apps they are using or through a systematic search of the app store. We sought to evaluate the most likely common apps that pregnant women are to encounter, which we did through a Google search [14]. Furthermore, to our knowledge, pregnancy apps have not previously been scored with the APPLICATIONS scoring system. App stores have an abundance of options, requiring consumers to sift through apps that may not be relevant (such as humorous apps for fake pregnancy tests), which can easily result in them feeling overloaded [20]. As most of the Google search results were written articles describing apps, erroneous and irrelevant apps from the app store are less likely to be included. This study is able to provide patients and providers with a curated list of rated apps and their features.

### Clinical Implications

The information found in this study may be particularly useful to women during the COVID-19 pandemic. Pregnant women often seek information on the internet and do not discuss much of what they find with their physicians [21]. Due to the pandemic, half of adults say that they or someone in their household has skipped seeking medical care due to anxiety about contracting COVID-19 [22]. Prenatal visits have needed to be rescheduled or modified, and many women are not satisfied with the level of information they are receiving through phone or video visits. The combination of these factors has likely led to the increase in pregnancy app downloads, which has occurred at a time when pregnant women have fewer touchpoints with doctors where false information could be corrected, emphasizing the need for high-quality and accurate pregnancy apps [4].

### Limitations

Given the dynamic nature of Google search results and the app store, several factors limit our analysis. This was evident in our search, as 12 of the apps initially identified were either no longer available or did not work by the time we tried to evaluate them. Additionally, we decided to use only the first two pages of Google search results as they account for most views. It is possible that other apps present on later pages were useful and comprehensive.

### Conclusions

The current method of app selection by the majority of women is to download the app and search the content themselves [12]. Although this may yield good results, it also means women must download multiple apps, as well as pay for many of these apps. On average, Lee showed that users download over three apps and are more likely to download a free app versus a paid app [8]. By using a Google search, we were able to identify many high-scoring apps that may be used during pregnancy. The identified rated apps can lessen the burden on pregnant women and providers to search for useful and comprehensive apps on their own. As telehealth continues to expand, more research is needed in the area of pregnancy app development.

## Appendix

Multimedia Appendix 1 Summary of evaluated and rated pregnancy apps.

## Abbreviations

APPLICATIONS: app comprehensiveness, price, privacy, literature used, in-app purchases, connectivity, advertisements, text search field, images/videos, other special features, navigation ease, subjective presentation
