# Supplementary material for: Mobile Health Apps for Pregnant Women: Systematic Search, Evaluation, and Analysis of Features
Source: J Med Internet Res. 2021 Oct 18;23(10):e25667. doi: 10.2196/25667 (PMC8561408; doi:10.2196/25667)
Supplement: Multimedia Appendix 1 [file jmir_v23i10e25667_app1.docx]

**Multimedia Appendix 1.** Summary of evaluated and rated pregnancy apps.

| App name | App icon | App comprehensiveness | Price | Privacy | Literature used | In-app purchases | Connectivity | Advertisements | Text search field | Images/videos | Other special features | Navigation ease | Subjective presentation | Total score |
| --- | --- | --- | --- | --- | --- | --- | --- | --- | --- | --- | --- | --- | --- | --- |
| BabyCenter | 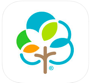 | 3 | 1 | 1 | 1 | 1 | 1 | 0 | 1 | 2 | 2^a,b,c,e,f^ | 1 | 1 | 15 |
| WebMD Pregnancy | 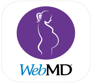 | 3 | 1 | 1 | 1 | 1 | 0 | 1 | 1 | 1 | 2^b,c,d,f,h,i^ | 1 | 1 | 14 |
| Ovia Pregnancy | 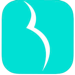 | 3 | 1 | 1 | 1 | 1 | 0 | 0 | 1 | 2 | 2^a,b,c,e,h^ | 1 | 1 | 14 |
| What to Expect | 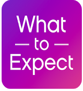 | 3 | 1 | 1 | 1 | 1 | 1 | 0 | 0 | 2 | 1^a^ | 1 | 1 | 13 |
| matriarc | 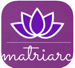 | 3 | 1 | 1 | 0 | 1 | 0 | 1 | 1 | 1 | 1^g,h^ | 1 | 1 | 12 |
| NSH BabyBump | 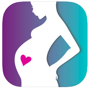 | 3 | 1 | 0 | 0 | 1 | 0 | 1 | 0 | 2 | 2^b,c,d,f,h^ | 1 | 1 | 12 |
| Pregnancy + | 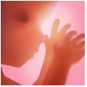 | 3 | 1 | 1 | 0 | 0 | 0 | 1 | 0 | 1 | 2^a,b,c,f,h^ | 1 | 1 | 11 |
| MommyMeds | 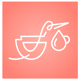 | 2 | 0 | 1 | 1 | 1 | 0 | 1 | 1 | 1 | 1^e^ | 1 | 1 | 11 |
| PelvicFloor | 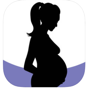 | 2 | 1 | 0 | 1 | 1 | 1 | 1 | 0 | 2 | 0 | 1 | 1 | 11 |
| Hello Belly | 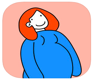 | 2 | 0 | 1 | 0 | 0 | 1 | 1 | 0 | 2 | 2^a,b,e,g,h^ | 0 | 1 | 10 |
| Glow | 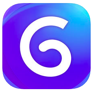 | 2 | 1 | 1 | 0 | 0 | 1 | 0 | 0 | 2 | 1^g^ | 1 | 1 | 10 |
| The Bump | 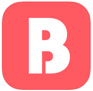 | 3 | 1 | 1 | 0 | 1 | 0 | 0 | 0 | 1 | 1^a,c,e,f^ | 0 | 1 | 9 |
| Glow Nurture | 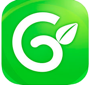 | 3 | 1 | 1 | 0 | 0 | 1 | 0 | 0 | 1 | 1^d,f^ | 0 | 1 | 9 |
| Kindara | 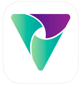 | 2 | 1 | 1 | 1 | 0 | 1 | 0 | 1 | 0 | 0 | 1 | 1 | 9 |
| Baby Pics | 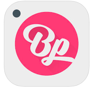 | 1 | 0 | 1 | 0 | 0 | 1 | 1 | 1 | 1 | 1^f^ | 1 | 1 | 9 |
| Contraction Timer | 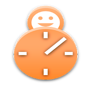 | 2 | 1 | 0 | 0 | 1 | 1 | 1 | 0 | 0 | 1^c^ | 1 | 1 | 9 |
| Sprout Pregnancy | 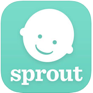 | 3 | 0 | 0 | 0 | 0 | 1 | 1 | 0 | 1 | 1^d,f,h^ | 0 | 1 | 8 |
| Totally Pregnant | 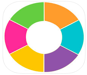 | 3 | 1 | 0 | 0 | 0 | 0 | 1 | 0 | 2 | 1^c,f,i^ | 0 | 0 | 8 |
| Fertility Friend | 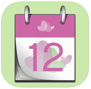 | 2 | 1 | 1 | 0 | 0 | 1 | 0 | 0 | 0 | 1^h^ | 1 | 1 | 8 |
| Expectful | 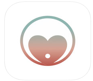 | 1 | 1 | 1 | 0 | 0 | 1 | 1 | 0 | 0 | 1^g^ | 1 | 1 | 8 |
| Who's Your Daddy | 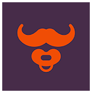 | 2 | 0 | 0 | 0 | 1 | 1 | 1 | 0 | 0 | 1^a,h^ | 1 | 1 | 8 |
| iPregnant | 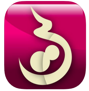 | 3 | 1 | 0 | 0 | 1 | 1 | 0 | 0 | 1 | 1^c,d^ | 0 | 0 | 8 |
| Mind the Bump | 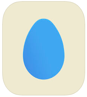 | 1 | 1 | 1 | 0 | 1 | 0 | 1 | 0 | 0 | 0 | 1 | 1 | 7 |
| Full Term App | 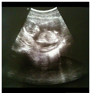 | 2 | 1 | 0 | 1 | 0 | 1 | 0 | 0 | 0 | 1^b,c,d^ | 0 | 1 | 7 |
| Tinybeans | 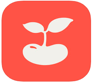 | 1 | 1 | 1 | 0 | 0 | 0 | 0 | 0 | 1 | 1^f,h^ | 1 | 1 | 7 |
| Pregnancy Assistant | 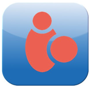 | 1 | 1 | 0 | 0 | 1 | 1 | 1 | 0 | 1 | 1^f^ | 0 | 0 | 7 |
| Oh Baby! Pregnancy workout | 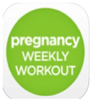 | 1 | 0 | 0 | 0 | 1 | 1 | 1 | 0 | 1 | 1^g^ | 1 | 0 | 7 |
| Pregnant Dad | 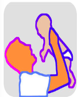 | 2 | 0 | 0 | 0 | 1 | 1 | 1 | 0 | 1 | 1^c,h^ | 0 | 0 | 7 |
| Positive Pregnancy with Andrew Johnson | 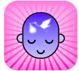 | 1 | 0 | 0 | 0 | 1 | 1 | 1 | 0 | 0 | 0 | 0 | 0 | 4 |

^a^Registry/baby shopping/products.

^b^Kick counter.

^c^Contraction timer.

^d^Tracker for mother (symptoms, weight).

^e^Safety information (nutrition, physical activity, medication).

^f^Journaling/photo uploads.

^g^Health and fitness (workout, meditation).

^h^Appointment tracker/checklist/calendar.

^i^Additional unique features.
